# Isolated torsion of the fallopian tube associated with hydrosalpinx in a 17‐year‐old sexually inactive girl: A case report

**DOI:** 10.1002/ccr3.4794

**Published:** 2021-09-15

**Authors:** Angelos Daniilidis, Sonia Charitidou, Stamatios Petousis, Chrysoula Margioula‐Siarkou, Anastasios Liberis, Konstantinos Dinas

**Affiliations:** ^1^ 2nd Department of Obstetrics and Gynaecology Aristotle University of Thessaloniki Thessaloniki Greece

**Keywords:** acute medicine, obstetrics and gynecology

## Abstract

Isolated tubal torsion is an unusual cause of acute abdominal pain in young sexually inactive patients. However, it should be still taken into account regarding the differential diagnosis of such conditions.

## Graphical abstract

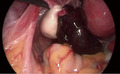

## 1 INTRODUCTION

Isolated tubal torsion is a rare cause of acute abdominal pain in women and is even less frequent in the patients younger than 18 years. We present the interesting case report of a tubal torsion accompanied with hydrosalpinx, diagnosed in a 17‐year‐old patient that was sexually inactive adolescent.

Hydrosalpinx is the condition in which the fallopian tube is filled with fluid as a result of total distal occlusion and abnormal distension of the ampullary portion.^1^ Isolated tubal torsion (ITT) is the rotation of the tube around its longitudinal axis, while the ovary and its blood flow remain unaffected.^2^, ^3^ Torsion of the right tube is much more commonly encountered than torsion of the left, which may be attributed to the fixation of the left tube in the left hemipelvis by the sigmoid colon. ITT is a rare cause of acute abdominal pain in women and is even less frequent in the patients younger than 18 years.^4^, ^5^

Although the pathophysiology of isolated torsion is not determined with certainty in many cases, several pathologic conditions have been described as risk factors. Pelvic inflammatory disease, hydrosalpinx, endometriosis, paratubal, para‐ovarian masses, hydatid cyst of Morgani, autonomic dysfunction of the fallopian tube, previous abdominal operations, history of peritonitis are some of the main causes of a tubal torsion.^6^, ^7^, ^8^, ^9^, ^10^, ^11^, ^12^, ^13^, ^14^, ^15^, ^16^, ^17^ Such pathologies are more frequently diagnosed in adults, while it is unusual to make a relative diagnosis in younger population that is rather less sexually active.

We present the interesting case report of a tubal torsion accompanied with hydrosalpinx, diagnosed in a 17‐year‐old patient that was sexually inactive adolescent with no previous abdominal surgeries.

## 2 CASE PRESENTATION

Α 17‐year‐old girl was admitted as an emergency to our gynae department reporting nausea, over ten episodes of vomiting, and convulsive pain to the left lower abdomen. Symptoms initiated 2 h before admission to hospital. Menstruation was rather abnormal, while the last regular gynecologic examination was totally normal 2 years ago. She had no history of sexual intercourses and also no history of any gynecological or any other abdominal surgery. On admission, blood pressure was 135/75 mmHg, pulse 90/min, body temperature was 36°C and oxygenated hemoglobin was normal. She had a BMI of 23.

**FIGURE 1:**
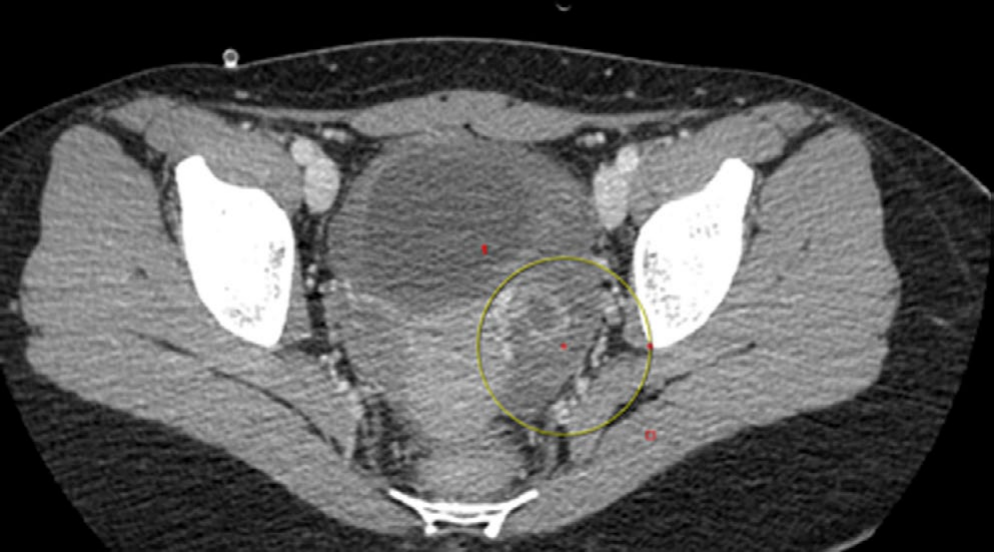
CT image: Presence of cystic mass surrounded by edematous stroma and engorged blood vessels

Clinical examination revealed normal bowel movements on auscultation, but also tense and sensitivity on abdominal palpation mainly to the left lower quadrant. No vaginal examination was performed, as the patient was virgo and there was no sign of vaginal bleeding. Peripheral blood test showed mild leukocytosis 11.100/μl and normal C‐reactive protein of 1.3 mg/L. The hemoglobin level was of 11.7 g/dl. Transabdominal ultrasound demonstrated a large unilocular cyst of about 8 cm in diameter in the left ovary with reduced vascularity. Based on clinical and ultrasound findings, there was a high suspicion of ovarian torsion and a computed tomography (CT) was asked in order to confirm or exclude the potential diagnosis of ovarian torsion. Decision for surgical treatment with laparoscopy was thereafter decided. [Figure 1].

**FIGURE 2:**
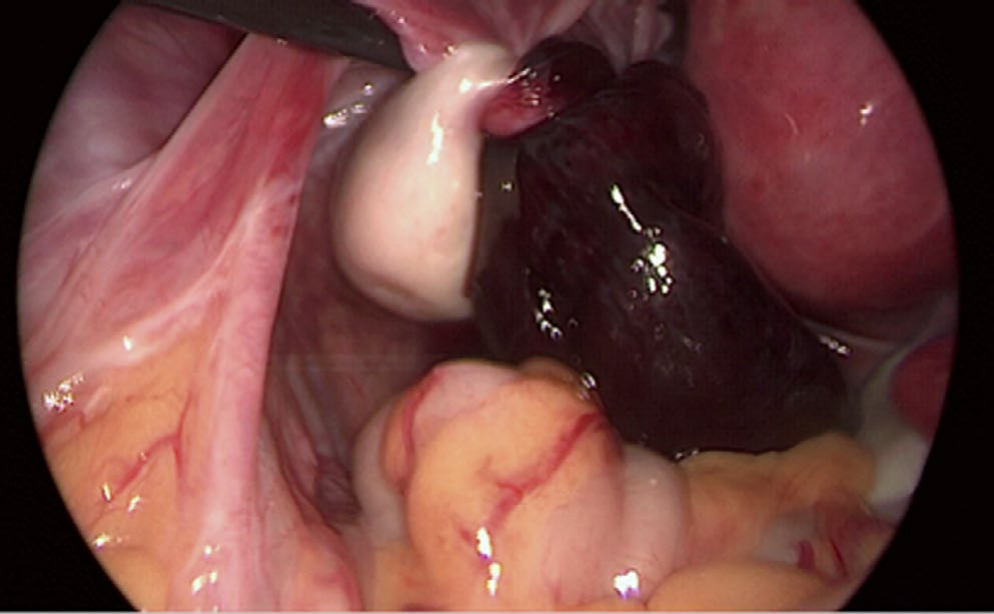
Intraoperative image of ischemic left fallopian tube

Laparoscopy was performed under general anesthesia. Four trocars were used: one of 12 mm diameter at the umbilicus and three peripheral of 5 mm to the lower abdomen. Intraoperative findings included a massively enlarged fimbrial funnel and a paraovarian cystic tumor with torsion of the distal part of the fallopian tube [Figure 2]. The tumor appeared infarcted, while inflammatory fluid was released after puncture. This invasions did not result in improvement of clinical condition. However, the other ovary was macroscopically normal and the rest of the abdominal cavity. No signs of possible infectious disease were identified.

In order to preserve ovary, the necrotic mass was resected at the level of corresponding mesosalpinx from the proximal to the distal end of the fallopian tube therefore performing a left salpingectomy. The cystic mass was afterward sent for pathological examination. Diagnosis was acute hemorrhagic necrosis, while hydrosalpinx was also histologically reported.

The patient recovered from the surgery without any complications and was discharged uneventfully onthe first postoperative day.

## 3 DISCUSSION

This case report describes the extremely rare diagnosis of an isolated tubal torsion with coexisting hydrosalpinx in a 17‐year‐old patient without history of sexual intercourses.

A possible explanation for ITTH in adolescents could be the presence of a congenital malformation of the tube in the peripubertal period. As the reproductive axis is stimulated between 9 and 14 years, menses may activate ovarian and tubal function, revealing a previously asymptomatic distal occlusion of the tube. An episode of asymptomatic pelvic inflammation near tubes may cause a distal occlusion, hydrosalpinx, and then torsion. Besides, torsion of the hydatid cyst of Morgani, located near the fimbriated end of the tubes, could also cause the pathologic process.

Diagnosis of ITT is usually difficult because symptoms are non‐specific and common with many other conditions.^7^ The typical presentation of ITTH is acute lower abdominal pain with nausea and vomiting, but no specific clinical feature allows with safety to distinguish this from torsion involving the whole adnexa. Absence of fever and normal C‐reactive protein levels may be helpful to make the differential diagnosis from appendicitis.

Regarding most common location, Boukaidi et al conducted a review of the literature and targeted reports published from 1999 to 2009 where 13 cases of ITTH in adolescents were reported.^3^ In their series, ITTH occurred on the left side in 9 of the 13 cases. This might suggest that ITTH occurs more frequently on the left tube although confirmation by a larger series of patients is needed.

Ultrasound is the imaging modality of choice as it is non‐invasive and avoids radiation exposure but diagnosis is not always definitive. Abdominal ultrasound showing the fallopian tubes as fluid‐filled tubular structures folded onto themselves to form a C or S shape and separated from ovaries is consistent with a diagnosis of hydrosalpinx. Color Doppler may be useful, but the presence of normal flow does not necessarily rule out torsion.^18^, ^19^, ^20^ Computerized tomography (CT) as well as magnetic resonance imaging (MRI) may be contributive as they may demonstrate characteristics such as abnormal fallopian tube, twisting of the adnexal pedicle, and a septal appearance of the fallopian tube dilated and fluid‐filled1.^18^, ^19^, ^20^, ^21^ However, the gold standard for confirming diagnosis is laparoscopy, with all relative advantages of minimally invasive procedure that permit quick recovery and minimal morbidity.^21^

Finally, gravity of clinical condition is associated with the duration and degree of torsion. Boukkaidi et al. proposed a classification of the tubal status by conducting salpingoscopy. Grades I and II would correspond to potentially salvageable fallopian tube, whereas grades III or more would require salpingectomy. According to this proposal, Grade I and II are treated by puncturing the hydrosalpinx and completing a detorsion. The correction of the distal occlusion is established by salpingoplasty few weeks later. In the contrary, grade III represents a compromised tube that indicates the necessity of salpingectomy.^2^

In conclusion, isolated tubal torsion associated with hydrosalpinx in children and sexually inactive adolescents is an extremely rare entity. Its presentation raises difficulties in the differential diagnosis. Ultrasonography with Doppler should be the primary imaging option; however, laparoscopy remains the gold standard I in both diagnosis and treatment.

## CONFLICT OF INTEREST

The authors declare no conflict of interest.

## AUTHOR CONTRIBUTIONS

All authors of the present manuscript have contributed significantly and their contribution justifies authorship. AD, SC, and SP write the initial draft. C M‐S provided significant language editing and revision to manuscript. AL and KD also revised critically the draft.

## ETHICAL APPROVAL

The authors report compliance with all ethical standards required.

## CONSENT

Informed consent was obtained from the patient.

## Data Availability

Data may be available for potential people interested in them.
